# Curcumin in Atherogenic Dyslipidemia: Linking Preclinical Mechanistic Insights to Clinical Outcomes

**DOI:** 10.3390/nu18142279

**Published:** 2026-07-11

**Authors:** Kamil Brodziński, Justyna Juszczyńska, Joanna Karbowska, Zdzislaw Kochan

**Affiliations:** 1 Laboratory of Nutritional Biochemistry, Department of Clinical Nutrition, Medical University of Gdansk, 80-211 Gdansk, Poland; kamil0699@gumed.edu.pl (K.B.); j.juszczynska@gumed.edu.pl (J.J.); 2 Department of Biochemistry, Medical University of Gdansk, 80-211 Gdansk, Poland

**Keywords:** curcumin, atherosclerosis, triglycerides, HDL-C, LDL-C, lipoprotein(a), lipid-lowering, phytochemicals, polyphenols, nutraceuticals

## Abstract

**Background/Objectives**: Atherogenic dyslipidemia is a major cardiometabolic risk factor characterized by elevated circulating triglycerides (TGs), reduced HDL-C, and increased levels of atherogenic lipoproteins. Curcumin, a polyphenolic compound considered the main bioactive component of turmeric (*Curcuma longa*), has attracted growing interest because of its potential lipid-modifying and anti-inflammatory properties. This scoping review aimed to evaluate evidence from randomized controlled trials (RCTs) on the efficacy of curcumin supplementation in the management of atherogenic dyslipidemia and to summarize current mechanistic evidence related to curcumin absorption, metabolism, and regulation of lipid homeostasis. **Methods**: A PRISMA-ScR-guided scoping review was performed across five databases (PubMed, Scopus, Web of Science, Cochrane Library, and Embase). RCTs evaluating curcumin supplementation in atherogenic dyslipidemia or related cardiometabolic conditions were systematically identified and synthesized. Mechanistic and preclinical evidence was identified through separate topic-specific searches of PubMed, Scopus, and Web of Science, supplemented by citation searching, and was synthesized narratively. **Results**: Twenty-two RCTs published between 2008 and 2025 were included. Most studies involved patients with cardiometabolic disorders, including type 2 diabetes mellitus with hyperlipidemia, metabolic syndrome, and polycystic ovary syndrome. Curcumin supplementation, administered in various formulations and dosages, showed overall favorable effects on plasma lipid profiles, particularly TGs and LDL-C, although the magnitude of these effects varied across studies. Mechanistic and preclinical evidence suggested that curcumin may modulate multiple pathways involved in lipid homeostasis, including intestinal cholesterol uptake, hepatic lipogenesis, cholesterol synthesis, fatty acid oxidation, bile acid metabolism, and reverse cholesterol transport. **Conclusions**: Current evidence suggests that curcumin may improve atherogenic lipid profiles through pleiotropic effects on lipid metabolism and cholesterol homeostasis. The clinical efficacy of curcumin appears to depend substantially on formulation-related bioavailability. Despite inter-study heterogeneity, curcumin shows potential as an adjunctive strategy for the management of atherogenic dyslipidemia and associated metabolic disorders.

## 1. Introduction

Atherogenic dyslipidemia—defined by elevated blood triglycerides (TGs), low high-density lipoprotein-cholesterol (HDL-C), and an abundance of small, dense low-density lipoprotein (LDL) particles—is a common, lifestyle-linked lipid pattern in contemporary Western populations. Roughly one in ten adults carries the atherogenic TG-high and HDL-low serum lipid profile, and the prevalence doubles among people with obesity, type 2 diabetes, or metabolic syndrome, markedly increasing cardiovascular risk in affected individuals. Because this lipid profile is usually an acquired, secondary dyslipidemia driven by modifiable lifestyle and metabolic factors—such as diet, excess weight, and insulin resistance—its associated cardiovascular risk can be substantially reduced by addressing those underlying causes. This insight raises the possibility that targeted dietary management or nutraceuticals could complement conventional pharmacological therapy.

**Figure 1:**
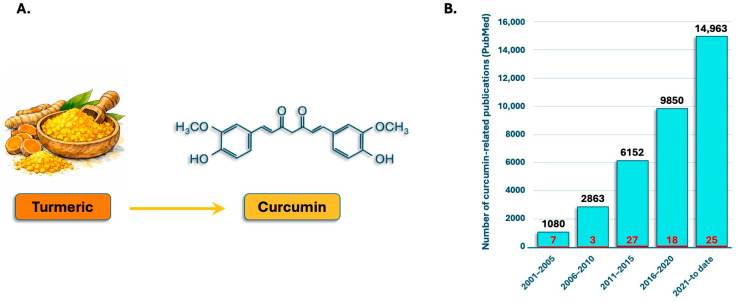
(**A**) Chemical structure of curcumin. (**B**) Number of publications on curcumin (black numbers) and on curcumin in the context of atherogenesis or atherogenic mechanisms (red numbers) by time period in the 21st century. The PubMed search for the red-numbered data used the terms “atherogenesis” or “atherogen*” (Source: PubMed, accessed on 1 February 2026). Schematic created by the authors (K.B., J.K. and Z.K.).

Natural plant-derived compounds have been used for medicinal purposes across many cultures for centuries. Historical and contemporary evidence documents their use in the Americas, Europe, and Asia, including within Ayurveda—the traditional system of Indian medicine—where preparations of *Curcuma longa* (turmeric) occupy a prominent place. Turmeric, which predominantly grows in tropical and subtropical climates, was introduced to Europe in the 13th century by Arab traders as a less costly substitute for saffron. Its common name is thought to derive from the Arabic word *kurkum*, meaning “yellow dye” or “saffron,” which also underlies the alternative designation “Indian saffron.” The discovery of curcumin, the principal polyphenolic constituent of turmeric (Figure 1A), dates back to the early 20th century, when Vogel and Pelletier isolated a yellow pigment from the rhizomes of *Curcuma longa* [1]. The chemical structure of curcumin was elucidated in 1910, and its chemical synthesis was achieved in 1913 by the Polish chemist Wiktor Lampe [2].

Against this historical and ethnomedical background, evidence-based medicine has long regarded the therapeutic claims of traditional herbal remedies with skepticism. However, an increasing body of research supports the pharmacological activity of selected plant-derived phytochemicals [3,4,5]. This shift is reflected in the growing scientific interest in curcumin since the early 2000s (Figure 1B). As a natural polyphenolic compound, curcumin can directly scavenge reactive oxygen species (ROS) [6]; it has also been reported to restore or enhance the activity (and, in some contexts, the expression) of antioxidant enzymes, including superoxide dismutase (SOD), catalase (CAT), and glutathione peroxidase (GPx) [7,8,9,10]. This antioxidant activity is thought to contribute to curcumin’s broad tissue-protective properties, including hepatoprotective, cardioprotective, and neuroprotective effects [7,8,10,11,12]. It is also implicated in curcumin’s anti-inflammatory and anti-atherogenic actions [9]. However, despite the marked increase in the number of publications on curcumin in recent years, those focusing on atherogenesis or atherogenic mechanisms remain comparatively scarce (Figure 1B).

The aim of this scoping review is to synthesize current evidence from randomized controlled trials (RCTs) on the efficacy of curcumin supplementation in the management of atherogenic dyslipidemia, with a focus on its effects on plasma lipids and atherogenic lipoproteins. This review also summarizes experimental and human data on the absorption and metabolism of curcumin in the intestine and liver and on strategies to enhance its bioavailability, examines evidence on the modulation of cytochrome P450 enzyme activities by curcumin, and discusses curcumin-mediated regulation of intestinal cholesterol absorption as well as its interaction with key signaling pathways and molecules involved in the regulation of hepatic lipid metabolism.

## 2. Methods

### 2.1. Study Design

This study was conducted as a scoping review in accordance with the PRISMA-ScR (Preferred Reporting Items for Systematic Reviews and Meta-Analyses Extension for Scoping Reviews) guidelines [13]. The review protocol was not registered. As part of the scoping review, we systematically identified and synthesized RCTs to establish the clinical evidence base. Findings from this systematic identification and synthesis, derived from included primary experimental studies, were incorporated into the evidence map. Two authors (K.B. and Z.K.) independently screened titles and abstracts and assessed full-text articles for eligibility; disagreements were resolved by discussion. Mechanistic and preclinical evidence, including in vitro, animal, pharmacokinetic, and relevant human mechanistic studies, was identified through separate topic-specific database searches and supplementary citation searching and was synthesized narratively by the authors.

### 2.2. Search Strategy

**Figure 2:**
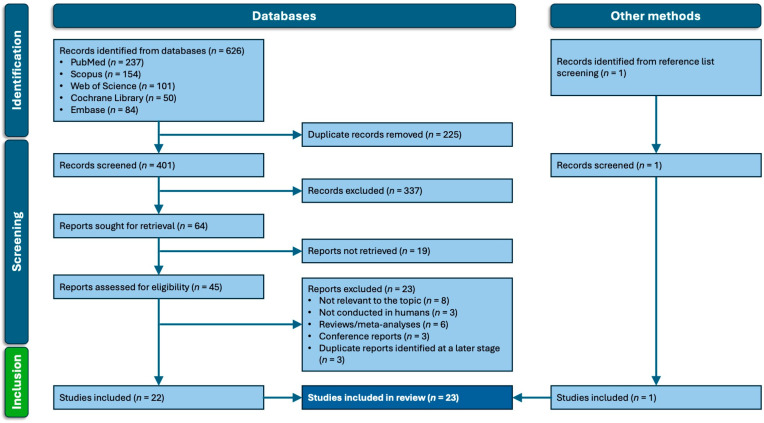
Flow diagram of the study selection process, showing identification of records through databases and reference-list screening. Schematic created by the authors (J.K., Z.K. and K.B.).

A comprehensive literature search was conducted in July 2025 in PubMed, Scopus, Web of Science, Cochrane Library, and Embase. The PubMed search strategy was as follows: (“dyslipidemias”[MeSH Terms] OR “dyslipidemia*”[Title/Abstract] OR “hyperlipidemia*”[Title/Abstract] OR “hyperlipoproteinemia*”[Title/Abstract] OR “hypercholesterolemia”[Title/Abstract] OR “hypertriglyceridemia*”[Title/Abstract]) AND (“curcumin*”[Title/Abstract] OR “turmeric”[Title/Abstract] OR “curcuma”[Title/Abstract] OR “curcuma longa”[Title/Abstract] OR “curcuminoid*”[Title/Abstract] OR “nanocurcumin”[Title/Abstract] OR “phytosomal curcumin”[Title/Abstract] OR “bisdemethoxycurcumin”[Title/Abstract] OR “demethoxycurcumin”[Title/Abstract]). Equivalent search strategies adapted to database-specific syntax were applied in Scopus, Web of Science, Cochrane Library, and Embase. The numbers of records retrieved were PubMed (*n* = 237), Scopus (*n* = 154), Web of Science (*n* = 101), Cochrane Library (*n* = 50), and Embase (*n* = 84). After duplicate removal, 401 records were screened (titles/abstracts). Full-text reports were retrieved and assessed for eligibility, and 22 studies were ultimately included from the database search. The study selection process is summarized in the flow diagram (Figure 2).

### 2.3. Eligibility Criteria

Eligibility criteria were predefined and structured according to the PICOS framework [14]. Population (P): Adults with dyslipidemia or cardiometabolic conditions characterized by elevated triglycerides and/or reduced HDL-C. Intervention (I): Curcumin supplementation or curcumin-containing formulations, administered alone or as adjunctive therapy. Comparator (C): Placebo, standard therapy, or other comparator interventions. Outcomes (O): Plasma triglycerides, HDL-C, LDL-C, total cholesterol, small dense LDL particles, and related atherogenic lipoprotein parameters. Study design (S): Randomized controlled trials (RCTs). No language restrictions were applied. Only peer-reviewed full-text articles were included. Preclinical studies, including in vitro and animal models, were identified separately and included narratively to provide mechanistic context.

### 2.4. Data Extraction

From each included RCT, the following data were extracted: study design, sample size, participant characteristics, curcumin formulation and dosage, intervention duration, comparator, lipid-related outcomes, and main findings. Two authors (K.B. and J.J.) independently extracted the data, and a third author (Z.K.) cross-checked all entries for accuracy; disagreements were resolved by discussion.

### 2.5. Methodological Appraisal

Given the scoping nature of this review, a formal quantitative risk-of-bias scoring system was not applied. Instead, the methodological characteristics of the included RCTs were assessed narratively, with attention to the following domains: randomization procedures, blinding, baseline comparability, intervention duration, sample size, and transparency of outcome reporting. This approach allowed a contextual evaluation of study robustness while maintaining the exploratory scope of the review.

### 2.6. Identification and Selection of Mechanistic and Preclinical Evidence

A separate, targeted literature search was conducted to identify mechanistic and preclinical studies relevant to the interpretation of the clinical findings. Because this component was intended to provide biological context rather than to constitute an independent systematic review, the search was organized as a series of topic-specific searches addressing the principal mechanisms discussed in Section 4, Section 5 and Section 6.

PubMed, Scopus, and Web of Science were searched using terms related to curcumin and its derivatives combined with terms describing individual mechanistic pathways. The curcumin search block included the following terms: “curcumin”, “turmeric”, “curcuma”, “curcuma longa”, “curcuminoid”, “nanocurcumin”, “phytosomal curcumin”, “bisdemethoxycurcumin”, and “demethoxycurcumin”. This block was combined separately with mechanistic terms related to intestinal absorption and bioavailability, curcumin metabolism, cytochrome P450 enzymes, NPC1L1-mediated cholesterol uptake, hepatic lipid metabolism, microRNAs, SIRT1, PPARα, ChREBP, SREBP-1, SREBP-2, fatty acid oxidation, cholesterol synthesis, bile acid metabolism, and reverse cholesterol transport. The targeted search strategies are provided in Supplementary File S1.

For example, the PubMed search addressing cytochrome P450-related mechanisms combined the curcumin search block with “cytochrome P450” in the title or abstract, whereas the search addressing intestinal cholesterol uptake combined the same curcumin block with “NPC1L1”. Equivalent searches were adapted to the syntax of Scopus and Web of Science. The searches primarily covered publications from January 2015 to July 2025 in order to identify recent mechanistic evidence. Earlier studies were also considered when they represented seminal experiments or were repeatedly cited as the primary source for a particular mechanism.

Titles and abstracts were screened for relevance to the mechanistic scope of the review. Full-text articles were examined when they reported experimental evidence concerning curcumin absorption, metabolism, transport, bioavailability, or regulation of pathways involved in lipid and lipoprotein homeostasis. Eligible evidence included in vitro studies, experiments in cell culture, animal studies, pharmacokinetic investigations, and relevant human mechanistic studies. Studies were selected for narrative inclusion when they directly supported, clarified, or qualified a mechanism discussed in relation to the clinical findings.

The database searches were supplemented by backward and forward citation searching. Reference lists of relevant reviews and primary experimental articles were screened, and additional publications were identified using PubMed’s “Similar articles” and “Cited by” functions. The mechanistic studies were not incorporated into the RCT evidence map and were not subjected to the same PRISMA-ScR study-selection process as the clinical studies. Instead, they were synthesized narratively to illustrate the biological plausibility of the clinical effects and to identify areas in which mechanistic evidence remains incomplete or inconsistent.

Mechanistic publications were prioritized when they: examined curcumin or a defined curcuminoid preparation; evaluated a pathway directly related to lipid absorption, hepatic lipid metabolism, cholesterol homeostasis, or atherogenic lipoprotein regulation; reported original experimental findings; and provided sufficient methodological detail to interpret the findings. Review articles were used primarily to identify relevant primary studies and to contextualize the evidence. Studies addressing unrelated pharmacological effects of curcumin were excluded from the mechanistic synthesis.

## 3. Clinical Evidence from Randomized Controlled Trials

### 3.1. Characteristics of Included Studies

A total of 23 RCTs were included in this scoping review (Table 1). The studies were published between 2008 and 2025, and their temporal distribution reflects increasing scientific interest in the potential role of curcumin in lipid management over the past decade. Only one study (4.4%) was published before 2014, whereas 12 studies (52.2%) were published between 2014 and 2019 and 10 studies (43.5%) were published between 2020 and 2025.

### 3.2. Geographic and Population Distribution

The included trials were conducted in seven countries: the United States, Australia, Indonesia, Egypt, Iran, Italy, and Turkey. These geographically diverse study populations may have differed in habitual dietary patterns, which could have influenced lipid responses. However, study location alone cannot be used to infer adherence to a specific dietary pattern. Although randomization likely reduced baseline dietary imbalance within individual trials, most studies did not standardize or systematically assess dietary intake during follow-up. Background diet may therefore have contributed to between-study heterogeneity, but its direction and magnitude cannot be determined from the available data.

The study populations predominantly consisted of individuals with underlying cardiometabolic disorders, most commonly type 2 diabetes mellitus with hyperlipidemia (T2DM/HL) (26%; *n* = 6), polycystic ovary syndrome (PCOS) (13%; *n* = 3), and metabolic syndrome (MetS) (13%; *n* = 3). Other populations included patients with atherosclerotic cardiovascular disease (ASCVD), individuals undergoing hemodialysis (HD), and participants with obesity.

Most studies included both male and female participants, whereas trials involving patients with PCOS exclusively recruited women. All participants were adults, typically between 30 and 65 years of age.

### 3.3. Sample Size

Most included trials were characterized by small- to medium-sized study populations: 12 studies (52.2%) enrolled fewer than 30 participants, 10 studies (43.5%) included between 30 and 60 participants, and only one study (4.4%) enrolled more than 60 participants. These findings indicate that the current evidence base is largely derived from relatively small clinical studies.

### 3.4. Curcumin Formulations and Dosage Regimens

The interventions differed with respect to curcumin formulation and dosage regimen. Conventional curcumin or turmeric preparations without a named bioavailability enhancer were used most frequently and were employed in 12 trials: these included curcumin powder, turmeric powder, turmeric extract, and curcuminoids. Bioavailability-enhanced formulations included phytosomal curcumin in five studies and nanocurcumin in two studies. Piperine or black pepper was co-administered in five studies, including one study that combined piperine with phytosomal curcumin.

Administered doses varied substantially across studies, ranging from 93.3 mg/day to 4.5 g/day. Lower doses were generally associated with bioavailability-enhanced formulations, whereas higher doses were more commonly used with conventional curcumin or turmeric preparations. Doses between 180 mg/day and 1.5 g/day were most frequently investigated.

Differences in curcumin formulation may be important when interpreting the variability of lipid responses across trials. Conventional curcumin, piperine-containing preparations, phytosomal curcumin, and nanoparticle formulations differ in solubility, intestinal uptake, and first-pass metabolism; therefore, administered dose alone may not accurately reflect systemic exposure to curcumin. These formulation-related factors are discussed below as potential contributors to heterogeneity in the clinical findings.

### 3.5. Formulation, Dose, and Systemic Exposure as Sources of Clinical Heterogeneity

The dose–exposure distinction is clinically relevant because some trials reporting reductions in circulating TGs, LDL-C, or total cholesterol used enhanced or piperine-containing preparations, whereas some studies using higher-dose conventional turmeric or curcumin showed smaller or inconsistent effects. Although these observations do not establish a direct formulation–response relationship, they support considering differences in intestinal absorption, first-pass metabolism, and systemic exposure when interpreting the trial findings.

**Figure 3:**
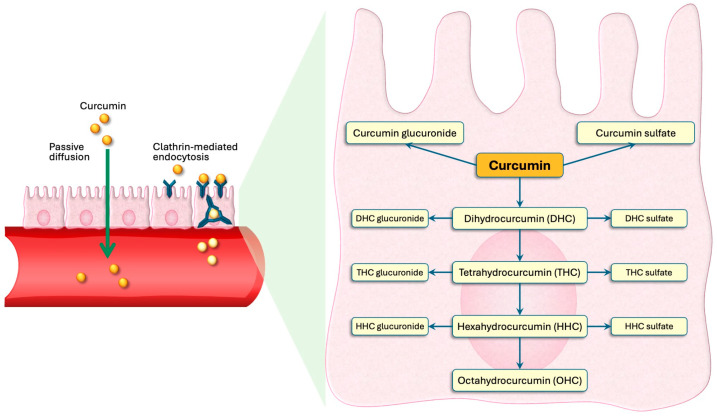
Intestinal absorption and metabolism of dietary curcumin. After oral administration, curcumin is absorbed from the intestinal lumen mainly via passive diffusion, with possible contribution from clathrin-mediated endocytosis. No specific intestinal transporter protein has been conclusively established as the dominant mediator of curcumin transport across enterocytes. In small intestinal enterocytes, curcumin undergoes extensive reductive and conjugative metabolism. Schematic created by the authors (J.K., K.B. and Z.K.), based on published evidence [43,45].

The clinical relevance of formulation heterogeneity reflects the pharmacokinetic limitations of curcumin. Poor oral bioavailability remains a major barrier to consistent systemic exposure and is largely attributable to low water solubility, chemical instability in aqueous environments, limited intestinal permeability, extensive intestinal and hepatic metabolism, and rapid elimination [38,39,40,41]. After oral administration, curcumin is absorbed mainly in the small intestine (Figure 3). Available evidence indicates that passive diffusion is a major route of enterocyte uptake, whereas endocytic pathways, including clathrin-mediated endocytosis, may contribute to uptake, particularly for some nanoparticle-based formulations [42,43]. No specific intestinal transporter has been conclusively established as the dominant mediator of curcumin transport. Bidirectional Caco-2 transport studies also suggest that active efflux is unlikely to be a major permeability-limiting mechanism for curcumin in this model [44].

Following uptake, curcumin undergoes rapid intestinal and hepatic biotransformation. Phase I reduction, mediated partly by cytochrome P450 enzymes, generates metabolites with varying degrees of biological activity, including dihydrocurcumin (DHC), tetrahydrocurcumin (THC), hexahydrocurcumin (HHC), and, less frequently, octahydrocurcumin (OHC) [46,47,48]. Among these, DHC and THC are considered principal active curcumin derivatives formed in humans [40,49,50]. These metabolites subsequently undergo phase II conjugation, mainly glucuronidation and sulfation, forming glucuronide and sulfate derivatives that are generally less biologically active than their unconjugated precursors [40,41,45,51]. As a result, conjugated forms predominate, whereas free curcumin is typically detected at very low concentrations, indicating limited systemic availability of the parent compound [40,45].

Several strategies have therefore been developed to improve intestinal absorption and systemic exposure of curcumin, including co-administration with piperine and the use of phytosomal, nanoparticle, micellar, and lipid-based delivery systems. Piperine, a major active constituent of black pepper, is among the most widely used bioavailability enhancers. In an early pharmacokinetic study, co-administration with piperine was reported to increase measured curcumin bioavailability by approximately 2000% [52], although more recent findings have questioned whether piperine substantially increases systemic exposure to unconjugated curcumin [40]. Piperine should not be regarded solely as an enhancer of intestinal absorption, because it may also modify intestinal and hepatic first-pass metabolism. Molecular-docking analyses suggest that piperine may interact with CYP3A4 and UDP-glucuronosyltransferases and thereby reduce oxidative metabolism and glucuronidation of curcumin, but the clinical relevance of these predicted interactions remains to be established [53]. Table 2 summarizes selected formulation strategies illustrating how curcumin bioavailability and systemic exposure may differ across preparations, including formulations used in the reviewed RCTs and other clinically relevant pharmacokinetic examples.

Absorption and bioavailability should be distinguished when interpreting the relative values summarized in Table 2. Absorption refers to gastrointestinal uptake, whereas bioavailability reflects the fraction of the administered dose that reaches systemic circulation in an active or measurable form. For curcumin, reported bioavailability is influenced not only by absorption but also by intestinal and hepatic metabolism, distribution, elimination, and analytical methodology.

A further distinction between free and total curcumin is essential when comparing pharmacokinetic data. Plasma free, unconjugated curcumin is generally considered the most biologically relevant circulating form and a key indicator of systemic bioavailability [58]. However, some studies quantify total curcumin after enzymatic hydrolysis of conjugated metabolites, which can substantially increase measured concentrations compared with non-hydrolyzed samples [58,59]. Differences in analytical methods and pharmacokinetic endpoints, including area under the concentration–time curve (AUC) and maximum plasma concentration (Cmax), therefore limit direct comparison of reported bioavailability values across curcumin formulations [60].

## 4. Mechanistic and Safety Considerations Relevant to Clinical Interpretation

### 4.1. Curcumin-Mediated Reduction in Intestinal Cholesterol Uptake via NPC1L1 Inhibition

One mechanism that may contribute to the LDL-C- and total cholesterol-lowering effects of curcumin observed in some clinical trials is reduced intestinal cholesterol uptake through downregulation of Niemann–Pick C1-like 1 protein (NPC1L1), the principal intestinal cholesterol transporter [61,62]. In human intestinal Caco-2 cells, curcumin reduced cholesterol uptake in a dose-dependent manner and decreased NPC1L1 mRNA and protein expression [61]. Subsequent studies showed that this effect is mediated, at least in part, by suppression of sterol regulatory element-binding protein 2 (SREBP-2), including reduced SREBP-2 expression, impaired DNA-binding activity, and inhibition of its site-1 protease (S1P)-dependent activation [62,63]. More recent studies indicate that curcumin-mediated *NPC1L1* suppression also involves hepatocyte nuclear factor 1α (HNF1α), another transcription factor regulating NPC1L1 [64,65]. Curcumin dose-dependently reduced cholesterol absorption and downregulated NPC1L1, SREBP-2, and HNF1α protein expression; moreover, silencing either transcription factor decreased *NPC1L1* expression and cholesterol absorption, supporting their role in NPC1L1 regulation [65]. Consistently, promoter activity assays showed that curcumin suppressed *NPC1L1* transcription by reducing the activity and nuclear abundance of SREBP-2 and HNF1α, thereby decreasing *NPC1L1* expression and cholesterol uptake in Caco-2 cells [64].

Animal studies provide supportive in vivo evidence for this mechanism. In high-fat diet-fed hamsters, which resemble humans in selected aspects of cholesterol metabolism and NPC1L1 tissue distribution, dietary curcumin supplementation (0.1% *w*/*w* for 12 weeks) downregulated intestinal NPC1L1 and components of the SREBP-2/HNF1α regulatory axis and increased fecal neutral sterol excretion, suggesting reduced intestinal cholesterol absorption [64,65].

Together, these in vitro and in vivo findings suggest that curcumin may decrease intestinal cholesterol uptake through attenuation of the NPC1L1/SREBP-2/HNF1α axis, providing a plausible mechanism for the LDL-C and TC reductions reported in some clinical studies. Thus, curcumin may contribute to improved systemic cholesterol homeostasis, although direct translation remains limited by differences in dose, formulation, species, and achieved intestinal and systemic exposure.

### 4.2. Interaction of Curcumin with Hepatic Triglyceride Metabolism

After intestinal absorption, curcumin and its metabolites reach the liver, where further metabolism occurs. Although circulating free curcumin levels are low, biologically active curcumin derivatives may affect hepatocellular lipid pathways, thereby enhancing lipid catabolism, reducing hepatic triglyceride accumulation, and decreasing very low-density lipoprotein (VLDL) secretion [49,50]. Table 3 summarizes signaling pathways affected by curcumin in the liver.

#### 4.2.1. Curcumin Activates AMPK Signaling and Reduces Hepatic Triglyceride Accumulation

Findings from human hepatic cell-line models indicate that curcumin promotes phosphorylation of AMP-activated protein kinase (AMPK), thereby increasing its activity [49,66]. Activated AMPK phosphorylates downstream metabolic targets, including the acetyl-CoA carboxylase (ACC) isoforms [67]. These effects have been reported in two human liver tumor-derived cell lines with distinct biological backgrounds: HepG2 cells, originally derived from hepatoblastoma and characterized by relatively hepatocyte-like features, and Hep3B cells, originally derived from hepatocellular carcinoma and characterized by hepatitis B virus positivity, p53 deficiency, and a more mesenchymal/fibroblast-like phenotype [81]. In these models, curcumin increased phosphorylation of AMPK and ACC, with ACC1 examined in HepG2 cells and ACC2 in Hep3B cells [49,66]. Because these are tumor-derived hepatic cell-line models, the findings should be interpreted as mechanistic support rather than direct evidence from normal human liver tissue.

Functionally, ACC1 catalyzes the first, rate-limiting step of hepatic fatty acid and TG synthesis, whereas ACC2 primarily regulates mitochondrial fatty acid β-oxidation. For both ACC isoforms, phosphorylation decreases enzymatic activity [67]. Thus, AMPK activation and subsequent phosphorylation of ACC1 and ACC2 would be expected to enhance fatty acid β-oxidation while suppressing fatty acid and TG synthesis. Consistent with these signaling changes, curcumin significantly reduced intracellular TG content in HepG2 hepatocytes [66]. In another HepG2 study, curcumin further lowered ACC1 enzymatic activity and protein abundance, thereby decreasing intracellular fatty acid and TG concentrations [68].

In vivo evidence supports these observations. In rats fed a high-fat, high-fructose diet (HFD + Fru) that initially induced hepatic steatosis and subsequently progressed to non-alcoholic steatohepatitis (NASH), intragastric curcumin administration (40 mg/kg for four weeks) similarly reduced hepatic ACC1 protein and lowered intrahepatic malonyl-CoA, the ACC-generated precursor for fatty acid synthesis [68]. Hepatic fatty acid and TG levels also decreased, and these changes coincided with reduced serum TG concentrations [68]. Likewise, in mice with steatohepatitis induced by a methionine- and choline-deficient (MCD) diet, a dietary mouse model of NASH, curcumin administration (100 mg/kg by oral gavage once daily for three weeks) increased hepatic AMPK and ACC phosphorylation and suppressed fat accumulation in the liver [7].

#### 4.2.2. Curcumin Modulates Hepatic Lipid Metabolism via MicroRNA-Mediated Pathways

MicroRNA networks are important post-transcriptional regulators of hepatic lipid synthesis and VLDL production [82]. Human microRNA-3666 (miR-3666) has been identified as a regulator of AMPK mRNA [66]. When elevated, miR-3666 negatively regulates AMPK by promoting AMPK mRNA degradation and translational repression. In HepG2 cells, miR-3666 overexpression reduced AMPK protein levels, whereas curcumin decreased miR-3666 abundance, suggesting an additional mechanism by which curcumin may enhance AMPK signaling [66].

Curcumin has also been reported to downregulate miR-22-3p in HepG2 cells and attenuate intracellular TG and cholesterol accumulation [69]. In hepatocytes, miR-22-3p directly targets the mRNAs encoding sirtuin 1 (SIRT1) and peroxisome proliferator-activated receptor α (PPARα) [70]. Both SIRT1 and PPARα contribute to transcriptional programs involved in hepatic fatty acid oxidation, triglyceride metabolism, and cholesterol homeostasis [71,72,83]. Thus, curcumin-induced downregulation of miR-22-3p may derepress SIRT1 and PPARα expression, thereby promoting hepatic lipid catabolism.

#### 4.2.3. Curcumin Upregulates the SIRT1–PPARα Axis and Enhances Hepatic Lipid Catabolism

The microRNA-mediated regulation described above implicates SIRT1 and PPARα as important downstream regulators of curcumin-associated hepatic lipid catabolism. In the liver, the NAD^+^-dependent deacetylase SIRT1 acts as a key sensor of fasting and cellular energy status and coordinates lipid metabolism by promoting fatty acid β-oxidation and suppressing lipogenesis [71]. SIRT1 modulates adaptive transcriptional responses through deacetylation of transcription factors and coactivators, including PPARα and peroxisome proliferator-activated receptor γ coactivator-1α (PGC-1α) [73]. Disruption of hepatic SIRT1 alters the expression of genes involved in lipid metabolism and impairs pathways governing lipid and cholesterol handling [71]. In mice, liver-specific knockdown of SIRT1 markedly increased intrahepatic free fatty acids and cholesterol and was associated with decreased expression of genes involved in fatty acid β-oxidation, cholesterol degradation, and cholesterol efflux [71]. In mice with MCD diet-induced steatohepatitis, curcumin (100 mg/kg, administered once daily for three weeks by oral gavage) elevated hepatic SIRT1 protein levels, which was associated with reduced lipogenesis and attenuated hepatic lipid accumulation [7].

PPARα is a nuclear receptor and transcription factor that regulates hepatic lipid catabolism, particularly fatty acid β-oxidation and TG turnover [72,83]. In HepG2 cells rendered steatotic by free fatty acid (FFA) stimulation, curcumin increased PPARα mRNA and protein levels [74]. Similar effects were observed in THLE-2 normal human liver cells subjected to FFA-induced steatosis, where curcumin elevated PPARα protein abundance [74]. Consistent with these findings, DHC, a reduced metabolite of curcumin, dose-dependently upregulated PPARα mRNA and protein expression in human liver-derived L-02 and HepG2 cell lines [50]. Additional preclinical evidence indicates that curcumin can enhance hepatic PPARα signaling, including higher PPARα mRNA levels and PPARα-dependent transcriptional activation of downstream targets [75]. In vivo, curcumin increased hepatic PPARα mRNA expression in LDL receptor-deficient mice fed an atherogenic diet, suppressed hepatic TG accumulation, and lowered circulating TGs, thereby preventing diet-induced hypertriglyceridemia [76]. Likewise, in a high-fat diet-induced NAFLD mouse model, curcumin elevated hepatic PPARα mRNA and protein levels, reduced hepatic lipid deposition, and improved the serum lipid profile by lowering TGs, TC, and LDL-C while raising HDL-C [74]. Curcumin also upregulated hepatic *CPT1A*, a PPARα target gene encoding carnitine palmitoyltransferase 1A (CPT1α), both in mouse liver in vivo and in hepatocytes in vitro, supporting enhanced mitochondrial fatty acid β-oxidation [74]. A recently proposed mechanism links curcumin-induced activation of the PPARα/CPT1α pathway to inhibition of the fat mass and obesity-associated (FTO) protein [74]. Because FTO normally demethylates and destabilizes PPARα mRNA, curcumin-mediated inhibition of FTO may preserve PPARα mRNA methylation, increase transcript stability, and thereby upregulate PPARα expression [74].

#### 4.2.4. Curcumin and Its Metabolites Suppress Hepatocellular Triglyceride Deposition by Downregulating ChREBP and SREBP-1

Hepatic de novo fatty acid synthesis and TG formation are largely regulated by two major lipogenic transcription factors: the glucose-responsive carbohydrate-responsive element-binding protein (ChREBP) and the insulin-responsive sterol regulatory element-binding protein 1 (SREBP-1). Together, these factors drive the expression of key lipogenic genes such as *ACACA* and *FASN* [84].

Evidence from animal studies indicates that curcumin can lower hepatic ChREBP expression in vivo. In MCD diet-fed mice, three-week curcumin treatment (100 mg/kg, once daily by oral gavage) reduced hepatic ChREBP protein levels [7]. Curcumin also decreased fatty acid synthase (FASN) protein levels in the liver and ameliorated hepatic steatosis [7], changes consistent with ChREBP downregulation and reduced lipogenic drive. In addition, curcumin and curcuminoids have been reported to activate hepatic AMPK [7,49,66]. Because AMPK can phosphorylate ChREBP and thereby lower its DNA-binding activity [77], curcumin may indirectly suppress ChREBP activity through AMPK-mediated signaling.

Curcumin-dependent AMPK activation may also promote phosphorylation of SREBP-1 and inhibit its proteolytic processing and transcriptional activity in the liver, thereby protecting against hepatic steatosis and hyperlipidemia [76]. In line with this mechanism, dietary curcumin supplementation in mice fed a high-fat/high-cholesterol diet activated hepatic AMPK signaling, as reflected by increased AMPK phosphorylation, and downregulated SREBP-1, ACC1, and FASN, suppressing hepatic lipid accumulation [78]. In the human HepG2 hepatic cell model, curcumin dose-dependently attenuated fatty acid-induced TG accumulation and, at an effective dose, reduced SREBP-1 and FASN mRNA levels [69]. DHC, the dihydro derivative of curcumin, likewise lowered TG content and decreased SREBP-1 mRNA in L-02 and HepG2 cells in a dose-dependent manner [50]. In rats, intragastric curcumin similarly downregulated hepatic SREBP-1 expression, accompanied by improvements in the circulating lipid profile: TG, TC, and LDL-C decreased, whereas HDL-C increased [79].

### 4.3. Curcumin Modulates Hepatic Cholesterol Homeostasis

#### 4.3.1. Curcumin May Attenuate Hepatic SREBP-2 Signaling and Cholesterol Synthesis

SREBP-2 regulates the expression of enzymes involved in cholesterol synthesis. Among these, 3-hydroxy-3-methylglutaryl-coenzyme A (HMG-CoA) reductase (HMGCR) is the pathway’s rate-limiting enzyme and is phosphorylated and thereby inhibited by AMPK [67]. Thus, curcumin-dependent activation of AMPK [7,49,66], followed by HMGCR phosphorylation, may reduce hepatic cholesterol synthesis.

Beyond AMPK-dependent inhibition of HMGCR activity, curcumin may also attenuate hepatic SREBP-2 signaling. In the human liver-derived cell line HepG2, curcumin dose-dependently decreased *SREBF2* mRNA, which encodes SREBP-2, and reduced the nuclear abundance of the mature active form of SREBP-2, thereby lowering SREBP-2–driven transcription [64]. Consequently, SREBP-2 target genes were downregulated. Across cellular and animal models, curcumin lowered hepatic expression of *HMGCR*, encoding HMG-CoA reductase, and *NPC1L1*, which encodes a transporter involved in biliary cholesterol reabsorption in the liver [64,65,69,76]. In HepG2 cells, curcumin also upregulated *CYP7A1*, which encodes cholesterol 7α-hydroxylase, the rate-limiting enzyme in cholesterol catabolism to bile acids [69]. By decreasing fatty acid-induced cholesterol synthesis and storage while favoring cholesterol disposal, curcumin prevented an increase in the hepatocellular total cholesterol pool [69].

Consistent with these findings, dietary curcumin (0.1% *w*/*w*) suppressed hepatic SREBP-2 in vivo, reduced hepatic TC, lowered serum TC and LDL-C, and increased HDL-C, thereby protecting high-fat diet-fed hamsters against hypercholesterolemia [64]. Similarly, in LDL receptor-deficient mice fed a high-cholesterol diet, curcumin (0.02% *w*/*w*) decreased hepatic cholesterol accumulation, lowered circulating LDL-C, and improved plasma atherogenic markers—including the HDL-C/TC ratio (%) and the apolipoprotein B (ApoB) to apolipoprotein A-I (ApoA-I) ratio—thus suppressing the development of atherosclerotic lesions [76].

#### 4.3.2. Curcumin Regulates LDLR–PCSK9 Signaling and LDL Clearance

Beyond its effects on hepatic cholesterol synthesis and disposal, curcumin has been reported to increase cell-surface low-density lipoprotein receptor (LDLR) abundance on hepatocytes by downregulating proprotein convertase subtilisin/kexin type 9 (PCSK9) expression [85]. PCSK9 is a key post-transcriptional regulator of LDLR: it binds the receptor, promotes its lysosomal degradation, and prevents its recycling to the cell surface [86]. Because LDLR mediates the uptake of circulating LDL particles from the blood, and the liver is the principal site of LDL clearance [87], curcumin-mediated augmentation of hepatic LDLR may lower systemic cholesterol levels and thereby further contribute to its anti-atherogenic effects.

A human study in healthy volunteers reported similar effects of curcumin on PCSK9, showing an approximately 10% reduction in plasma PCSK9 concentrations after one-week supplementation with 129 mg/day of micellar curcumin [88]. However, in moderately hyperlipidemic patients, six-week supplementation with 294 mg/day of micellar curcumin did not affect plasma PCSK9 concentrations or blood lipids [88]. This finding contrasts with other studies in which longer-term curcumin supplementation improved lipid profiles and leaves open the question of the potential clinical value of curcumin-mediated PCSK9 lowering in hyperlipidemia.

### 4.4. Safety and Curcumin–Drug Interaction Considerations Relevant to Clinical Translation

Oral curcumin intake is generally considered safe within established regulatory intake limits. The U.S. Food and Drug Administration (FDA) has raised no questions regarding the GRAS conclusion for curcumin from turmeric for specified food uses [89]. The Joint FAO/WHO Expert Committee on Food Additives (JECFA) established an acceptable daily intake (ADI) of 0–3 mg/kg body weight, whereas the European Food Safety Authority (EFSA) supported an ADI of 3 mg/kg body weight/day for curcumin as a food additive [90,91], corresponding to approximately 210 mg/day for a 70 kg adult. However, these ADI values refer to intake from food additive use and should not be interpreted as a general safety threshold for high-dose curcumin supplementation.

In a dose-escalation study in healthy volunteers, single oral doses of a standardized curcuminoid formulation ranging from 500 to 12,000 mg were associated with minimal toxicity [92]. Nevertheless, these single-dose findings should not be extrapolated to long-term safety, particularly because curcumin supplements vary in dose, bioavailability, duration of use, and co-ingredients such as piperine. Adverse effects have occasionally been reported, including liver injury associated with curcumin supplementation [93]. Moreover, curcumin may disrupt systemic iron metabolism, suggesting that individuals with iron deficiency and/or anemia should use curcumin-containing supplements with caution [94].

In addition to these general safety considerations, curcumin–drug interactions may be relevant to clinical translation. Curcumin not only undergoes metabolism by cytochrome P450 enzymes involved in xenobiotic and drug metabolism but may also modulate their activity. Depending on the CYP isoform and experimental conditions, including concentration and duration of exposure, curcumin has been reported to act as an inhibitor or inducer, potentially affecting its own pharmacokinetics and the disposition of co-administered drugs [95]. This issue may be particularly relevant in patients with atherogenic dyslipidemia, who frequently receive lipid-lowering therapy. For example, simvastatin, lovastatin, and atorvastatin are metabolized predominantly by CYP3A4, whereas fluvastatin is metabolized mainly by CYP2C9 [96]. Inhibition of these enzymes could theoretically increase statin exposure and the risk of concentration-dependent adverse effects, whereas induction could reduce drug exposure and therapeutic efficacy. CYP-mediated interactions may therefore represent a potential source of variability in trials evaluating curcumin as an adjunct to standard lipid-lowering treatment. However, most evidence for curcumin-mediated CYP modulation derives from in vitro or preclinical studies, and clinically significant interactions at customary supplemental doses have not been established. These findings should therefore be interpreted as mechanistically plausible rather than clinically confirmed. Supplementary File S2 summarizes the reported effects of curcumin on selected CYP isoforms, together with their physiological and pharmacological functions.

### 4.5. Linking Preclinical Evidence to Clinical Outcomes

**Figure 4:**
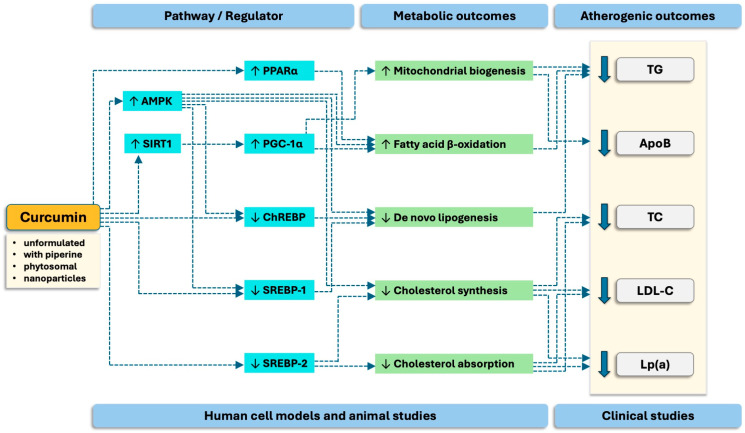
Schematic overview linking evidence from preclinical human cell models and animal studies to clinical lipid and atherogenic lipoprotein outcomes of curcumin. Cellular models and animal studies suggest that curcumin may modulate hepatic lipid metabolism and intestinal cholesterol handling through pathways involving AMPK, SIRT1, PGC-1α, PPARα, ChREBP, and SREBP-regulated processes, with potential downstream relevance to clinical changes in TG, TC, LDL-C, ApoB, and Lp(a). These mechanisms support the biological plausibility of lipid-related effects observed in clinical studies but remain to be confirmed as causal mediators in humans. Upward arrows (↑) indicate activation, upregulation, or an increase, whereas downward arrows (↓) indicate inhibition, downregulation, or a decrease. Dashed arrows indicate proposed directional links supported by preclinical evidence or biological plausibility and do not imply confirmed causal relationships in humans. Schematic created by the authors (J.K. and Z.K.).

Preclinical evidence provides biological plausibility for the lipid-modifying effects of curcumin observed in some clinical studies, but it does not establish direct causality in humans. In experimental models, curcumin affects several processes relevant to circulating lipid concentrations (Figure 4).

The strongest mechanistic support for TG lowering comes from diet-induced steatosis and dyslipidemia models, in which curcumin shifted hepatic lipid metabolism from lipogenesis toward lipid catabolism [7,68,74,76,78,79]. These effects are directionally consistent with the reductions in circulating TGs reported particularly in RCTs involving subjects with NAFLD, metabolic syndrome, or type 2 diabetes mellitus with hyperlipidemia.

For LDL-C and TC, the most directly relevant preclinical mechanism is curcumin-mediated reduction in intestinal cholesterol uptake, supported by Caco-2 cell studies and high-fat diet-fed hamster models [61,62,63,64,65]. Additional hepatic mechanisms, including decreased cholesterol synthesis, increased cholesterol disposal, and modulation of LDL uptake [64,69,76,85], may also contribute to improved cholesterol handling and are consistent with the LDL-C, TC, and selected atherogenic lipoprotein marker reductions observed in some RCTs. However, these mechanisms have not been confirmed as causal mediators in human supplementation trials.

Although the preclinical findings broadly agree with favorable lipid responses reported in some human studies, clinical effects remain heterogeneous. Animal studies generally used controlled atherogenic diets, genetically or diet-induced disease models, and curcumin exposures expressed as dietary percentages or body weight-adjusted doses, which are not directly comparable with clinical supplementation regimens. Human trials also differ in formulation, dose, duration, background diet, comorbidities, concomitant lipid-lowering therapy, and achieved systemic exposure. Moreover, most clinical trials assessed circulating lipid outcomes without measuring the mechanistic pathways implicated in cell and animal studies. Thus, it remains unclear whether the experimentally observed effects mediate lipid changes in humans, and differences in species, metabolism, formulation, and bioavailability limit direct translation.

## 5. Final Remarks

### 5.1. Limitations

The studies described in this review have limitations that warrant careful consideration. Differences in the reported bioavailability of curcumin formulations, as summarized in Table 2, can be attributed to several factors related to study design. One of the most important is sample size: smaller cohorts tend to yield greater variability, whereas larger samples provide more reliable and precise estimates. The selection of participants may also influence the findings, as factors such as age, sex, genetic variability, and underlying health conditions can affect the observed outcomes. In addition, the analytical methods used to measure curcumin concentrations may contribute to variability in the results. High-performance liquid chromatography (HPLC) and liquid chromatography–mass spectrometry (LC–MS) are commonly employed to separate, identify, and quantify curcumin and its metabolites; however, these techniques differ in their sensitivity, specificity, and overall analytical performance, which can lead to discrepancies in the reported bioavailability.

### 5.2. Future Research Directions

Future work should focus on developing optimized curcumin delivery systems and novel formulations to overcome its poor bioavailability. Hard endpoints such as cardiovascular events, as well as the safety of combining curcumin with other lipid-lowering drugs, remain untested and will require larger and longer RCTs. Moreover, curcumin’s ability to lower plasma PCSK9 concentrations, demonstrated in healthy volunteers, warrants further clinical studies in hyperlipidemic patients to determine its efficacy in the treatment of atherogenic dyslipidemia.

## 6. Conclusions

Taken together, the available evidence highlights curcumin’s multilevel regulatory effects on lipid metabolism and supports its potential role in the management of atherogenic dyslipidemia. Its anti-atherogenic actions appear to be mediated, at least in part, through modulation of hepatic lipid metabolism, including suppression of TG and cholesterol synthesis, as well as VLDL secretion.

Evidence from randomized controlled trials further suggests that curcumin supplementation may improve circulating lipid parameters in patients with atherogenic dyslipidemia and related cardiometabolic disorders, including metabolic syndrome and type 2 diabetes mellitus with hyperlipidemia. Reported effects include reductions in circulating TGs, TC, and LDL-C, accompanied by modest increases in HDL-C. However, the available clinical evidence remains limited by substantial heterogeneity in curcumin formulations, dosage regimens, and study populations, as well as by the predominance of relatively small clinical trials.

Despite its limited oral bioavailability, curcumin remains a promising adjunctive strategy for the management of atherogenic dyslipidemia and associated metabolic disturbances. Nevertheless, larger and longer-term RCTs employing standardized formulations and clinically relevant lipid-related and cardiovascular endpoints are needed to better define its therapeutic efficacy, safety profile, and optimal dosing strategies.

## Figures and Tables

**Table 1:** Effects of curcumin supplementation on circulating lipids and atherogenic lipoproteins in randomized controlled trials.

Author, Year	Study Design	Intervention	Duration	Outcomes vs. Control (Placebo) or Baseline [% Change]					
				TGs	HDL-C	LDL-C	TC	ApoB	Lp(a)
Nada et al., 2025 [15]	RCT, DB, PG (T2DM/HL)	Turmeric curcumin 1.1 g/d + black pepper 5 mg/d (*n* = 20) vs. B	12 wk	−27.6 **	−5.1	−13.4 **	−12.1 **	ND	ND
Dastani et al., 2023 [16]	RCT, DB, PG (T2DM/ASCVD)	Nanocurcumin 80 mg/d (*n* = 27) vs. B	12 wk	−10.2	−26.1 **	−14.5 **	−6.8 *	ND	−2.23 *
Laffin et al., 2023 [17]	RCT, SB, PG (ASCVD)	Turmeric curcumin 4.5 g/d + piperine 30 mg/d (*n* = 25) vs. B	4 wk	+0.3	+1.4	−1.3	−0.8	ND	ND
Atakan et al., 2022 [18]	RCT, PG (BMI > 25 kg/m^2^)	Turmeric curcumin 4 g/d (*n* = 35) vs. B	8 wk	−24.8	−36.2	−24.6	−24	ND	ND
Sangouni et al., 2022 [19]	RCT, DB, PG (MetS)	Curcumin 200 mg/d (*n* = 22) vs. B	12 wk	−25.8	+31.3	−15.8	−14.3	ND	ND
Darmian et al., 2021 [20]	RCT, SB, PG (T2DM/HL)	Turmeric curcumin 2.1 g/d (*n* = 11) vs. B	8 wk	−2.12	+13.6	−3.6	−2.7	ND	ND
Cicero et al., 2020 [21]	RCT, DB, PG (NAFLD)	Phytosomal curcumin 400 mg/d + piperine 16 mg/d (*n* = 40) vs. B	8 wk	−18.4 *	+10.0 *	−4.31	−4.15	ND	ND
Jamilian et al., 2020 [22]	RCT, DB, PG (PCOS)	Curcumin 500 mg/d (*n* = 24) vs. B	12 wk	−5.87	+3.54	−13.4	−8.6	ND	ND
Afshar et al., 2020 [23]	RCT, PG (HD)	Nanocurcumin 120 mg/d (*n* = 27) vs. B	12 wk	−1.25	+3.05	−0.13	−0.06	ND	ND
Asan et al., 2020 [24]	RCT, PG (PCOS)	Curcumin 93.3 mg/d (*n* = 15) vs. B	8 wk	−10.7	−4.64	−6.9	−6.67	ND	ND
Adab et al., 2019 [25]	RCT, DB (T2DM/HL)	Powdered rhizome of turmeric 2.1 g/d (*n* = 39) vs. B	8 wk	−21.9	+4.43	−8.89	+0.65	−1.78	ND
Adibian et al., 2019 [26]	RTC, DB, PG (T2DM/HL)	Curcumin 1.5 g/d (*n* = 21) vs. B	10 wk	−12.1	0	−3.6	−2.4	ND	ND
Sohaei et al., 2019 [27]	RCT, DB, PG (PCOS)	Turmeric curcumin 500 mg/d (*n* = 27) vs. B	6 wk	+5.61	+3.7	+3.4	−1.8	ND	ND
Thota et al., 2019 [28]	RCT, DB, PG (T2DM/HL)	Phytosomal curcumin 180 mg/d (*n* = 20) vs. B	12 wk	−0.79	0	−1.6	+1.92	ND	ND
Panahi et al., 2019 [29]	RCT (NAFLD)	Phytosomal curcumin 300 mg/d (*n* = 36) vs. B	8 wk	−21.3	−3.98	−32	ND	ND	ND
Ferguson et al., 2018 [30]	RCT, DB, PG (HC)	Phytosomal curcumin 200 mg/d (*n* = 18) vs. B	4 wk	−0,69	−3.73	−2.56	−2.31	ND	ND
Panahi et al., 2017 [31]	RCT, DB, PG (T2DM/HL)	Curcuminoids 1 g/d + piperine 10 mg/d (*n* = 50) vs. B	12 wk	−10.6	+3.82	−4.86	−10.1	ND	−16.1 **
Kocher et al., 2016 [32]	RCT, XO, DB (MHL)	Curcuminoids 294.2 mg/d (*n* = 42) vs. C	6 wk	−7.88	+0.18	−0.74	+1.15	ND	ND
Panahi et al., 2016 [33]	RCT (NAFLD)	Phytosomal curcumin 200 mg/d (*n* = 50) vs. C (*n* = 52)	8 wk	−19.5	−3.16	−22.8	−20.6	ND	ND
Amin et al., 2015 [34]	RCT, DB, PG (MetS)	Turmeric powder 2.4 g/d (*n* = 63) vs. B	8 wk	−6.84	+4.72	−5.13 **	−6.57 *	ND	ND
Lee & Lim 2015 [35]	RCT, PG (BDLD)	Turmeric extract 1.4 g/d (*n* = 21) vs. B	12 wk	0	+6.8	0	0	ND	ND
Panahi et al., 2014 [36]	RCT, DB, PG (MetS)	Curcuminoids 1 g/d + piperine 10 mg/d (*n* = 50) vs. B	8 wk	−8	+17.3	−13.1	−11.1	ND	−9.76
Alwi et al., 2008 [37]	RCT, DB (ACS)	Curcumin 180 mg/d (*n* = 15) vs. C (*n* = 26)	1 y	−18.5	+8.2	−10.1	−7.7	ND	ND

ACS, acute coronary syndrome; ApoB, apolipoprotein B; ASCVD, atherosclerotic cardiovascular disease; B, baseline; BDLD, borderline dyslipidemia; C, control (placebo); DB, double-blind; HC, hypercholesterolemia; HD, hemodialysis; HDL-C, high-density lipoprotein cholesterol; HL, hyperlipidemia; LDL-C, low-density lipoprotein cholesterol; Lp(a), lipoprotein(a); MetS, metabolic syndrome; MHL, moderate hyperlipidemia; NAFLD, nonalcoholic fatty liver disease; ND, not determined; PCOS, polycystic ovary syndrome; PG, parallel-group; RCT, randomized controlled trial; SB, single-blind; T2DM, type 2 diabetes mellitus; T2DM/HL, type 2 diabetes mellitus with hyperlipidemia; TC, total cholesterol; TGs, triglycerides; XO, crossover; * *p* < 0.01; ** *p* < 0.001. Table created by the authors (K.B., J.J. and Z.K.) based on data from the studies cited in each row (see “Author, Year” column).

**Table 2:** Selected bioavailability-related formulation strategies relevant to interpreting curcumin clinical studies.

Curcumin Preparation or Strategy	Reported Bioavailability Relative to Unformulated Curcumin	Main Mechanism Relevant to Clinical Interpretation	References
Unformulated curcumin	Baseline; low systemic availability	Poor water solubility, extensive intestinal and hepatic metabolism, and rapid elimination	[40,54]
Curcumin with piperine	2000% increase in relative bioavailability in humans; 154% increase in rats	Enhanced intestinal absorption and inhibition of intestinal/hepatic glucuronidation, thereby increasing systemic exposure	[52]
Colloidal nanoparticle curcumin dispersion/THERACURMIN	27.3-fold higher AUC in humans; 42.8–44-fold higher AUC in rats	Improved aqueous dispersibility and intestinal absorption through colloidal nanoparticle formulation	[55]
Nanoemulsion curcumin formulated with polyethylene glycols (PEGs)	Approximately 10.5-fold greater AUC and 39.4-fold higher Cmax than curcumin suspension in mice	Increased curcumin solubility and gastrointestinal absorption through PEG-based solubilization, surfactant-mediated enhancement of intestinal permeability	[56]
Micellar curcumin	185-fold higher AUC in all subjects; 277-fold in women and 114-fold in men	Improved aqueous solubilization through micellar formulation, increasing systemic exposure	[57]
Liquid droplet micromicellar curcumin formulation	Approximately 94-fold greater total-curcumin AUC at an equal product mass; approximately 522-fold greater AUC after normalization per milligram of administered curcumin	Micromicellar solubilization improves gastrointestinal absorption and systemic exposure	[38]

AUC, area under the plasma concentration–time curve; Cmax, maximum plasma concentration. Values are reported as described in the cited studies and should not be interpreted as directly comparable across formulations because of differences in study design, analytical methods, and pharmacokinetic endpoints. Table created by the authors (J.K., Z.K., K.B. and J.J.) based on data from the studies cited in each row (see “References” column).

**Table 3:** Curcumin-modulated hepatic lipid metabolism signaling pathways and their downstream processes in human cell models and animal studies.

Pathway/Regulator	Curcumin’s Effects	Key Targets	Metabolic Outcome
AMPK	↑ AMPK activation (phosphorylation) ↑ AMPK signaling (downregulation of miR-3666, a negative regulator of AMPK)	ACC1 (inactivated by phosphorylation) ACC2 (inactivated by phosphorylation) HMGCR (inactivated by phosphorylation)	↓ De novo lipogenesis (fatty acid and triglyceride synthesis) ↑ Fatty acid β-oxidation ↓ Cholesterol synthesis
SIRT1/PGC-1α	↑ SIRT1 signaling (downregulation of miR-22-3p, a negative regulator of SIRT1) ↑ SIRT1 protein levels	PGC-1α (activated by deacetylation) *CPT1A*, *ACOX1*, *ACADM*, *ACADS* (upregulated)	↑ Mitochondrial biogenesis ↑ Fatty acid β-oxidation
PPARα	↑ PPARα expression	*CPT1A*, *ACOX1*, *ACADM* (upregulated)	↑ Peroxisomal and mitochondrial fatty acid β-oxidation
ChREBP	AMPK-dependent phosphorylation of ChREBP diminishes its DNA-binding and transcriptional activity ↓ ChREBP protein levels	*ACACA*, *FASN* (downregulated)	↓ Carbohydrate-driven fatty acid and triglyceride synthesis
SREBP-1	↓ *SREBF1* expression AMPK-dependent phosphorylation of SREBP-1 that blocks its processing and nuclear translocation	*ACACA*, *FASN* (downregulated)	↓ De novo lipogenesis (fatty acid and triglyceride synthesis)
SREBP-2	↓ *SREBF2* expression ↓ processing/nuclear SREBP-2	*HMGCR*, *NPC1L1* (downregulated)	↓ Cholesterol synthesis and biliary cholesterol reabsorption

*ACACA*, the gene encoding acetyl-CoA carboxylase 1; *ACADM*, the gene encoding medium-chain acyl-CoA dehydrogenase; *ACADS*, the gene encoding short-chain acyl-CoA dehydrogenase; ACC, acetyl-CoA carboxylase; AMPK, AMP-activated protein kinase; ChREBP, carbohydrate-responsive element-binding protein; FASN, fatty acid synthase; *FASN*, the gene encoding fatty acid synthase; HMGCR, 3-hydroxy-3-methylglutaryl-coenzyme A (HMG-CoA) reductase; *HMGCR*, the gene encoding HMG-CoA reductase; NPC1L1, Niemann–Pick C1-like 1; PGC-1α, peroxisome proliferator-activated receptor γ coactivator-1α; PPARα, peroxisome proliferator-activated receptor α; SIRT1, sirtuin 1; *SREBF1*, the gene encoding SREBP-1; *SREBF2*, the gene encoding SREBP-2; SREBP, sterol regulatory element-binding protein. ↑, increase, activation, or upregulation; ↓, decrease, inhibition, or downregulation. Table created by the author (J.K.) based on data from [7,49,50,64,65,66,67,68,69,70,71,72,73,74,75,76,77,78,79,80].

## Data Availability

No new data were created or analyzed in this study. Data sharing is not applicable to this article.

## Supplementary Materials

The following supporting information can be downloaded at: https://www.mdpi.com/article/10.3390/nu18142279/s1, File S1: Targeted search strategies for identification and selection of mechanistic and preclinical evidence; File S2: Curcumin-induced modulation of cytochrome P450 isoforms and their functions. References [97,98,99,100,101,102,103,104,105,106,107,108] are cited in the supplementary materials”) or in the main text.

## Abbreviations

The following abbreviations are used in this manuscript:

*ACACA*	The gene encoding acetyl-CoA carboxylase 1
*ACADM*	The gene encoding medium-chain acyl-CoA dehydrogenase
*ACADS*	The gene encoding short-chain acyl-CoA dehydrogenase
ACC	Acetyl-CoA carboxylase
ACS	Acute coronary syndrome
AMP	Adenosine monophosphate
AMPK	AMP-activated protein kinase
ApoA-I	Apolipoprotein A-I
ApoB	Apolipoprotein B
AUC	Area under the plasma concentration–time curve
CAT	Catalase
ChREBP	Carbohydrate-responsive element-binding protein
Cmax	Maximum plasma concentration
DHC	Dihydrocurcumin
FASN	Fatty acid synthase
*FASN*	The gene encoding fatty acid synthase
FFA	Free fatty acid
GPx	Glutathione peroxidase
HC	Hypercholesterolemia
HDL	High-density lipoprotein
HDL-C	High-density lipoprotein cholesterol
HHC	Hexahydrocurcumin
HL	Hyperlipidemia
HMG-CoA	3-Hydroxy-3-methylglutaryl-coenzyme A
HMGCR	HMG-CoA reductase
*HMGCR*	The gene encoding HMG-CoA reductase
HNF1α	Hepatocyte nuclear factor 1α
LDL	Low-density lipoprotein
LDL-C	Low-density lipoprotein cholesterol
LDLR	Low-density lipoprotein receptor
MCD	Methionine- and choline-deficient
NASH	Non-alcoholic steatohepatitis
NPC1L1	Niemann–Pick C1-like 1
OHC	Octahydrocurcumin
PCSK9	Proprotein convertase subtilisin/kexin type 9
PGC-1α	Peroxisome proliferator-activated receptor γ coactivator-1α
PPARα	Peroxisome proliferator-activated receptor α
SIRT1	Sirtuin 1
SOD	Superoxide dismutase
*SREBF1*	The gene encoding SREBP-1
*SREBF2*	The gene encoding SREBP-2
SREBP	Sterol regulatory element-binding protein
T2DM	Type 2 diabetes mellitus
TC	Total cholesterol
THC	Tetrahydrocurcumin
VLDL	Very low-density lipoprotein
