# Supplementary material for: Curcumin in Atherogenic Dyslipidemia: Linking Preclinical Mechanistic Insights to Clinical Outcomes
Source: Nutrients. 2026 Jul 11;18(14):2279. doi: 10.3390/nu18142279 (PMC13414635; doi:10.3390/nu18142279)
Supplement: Supplementary file 1 [file nutrients-18-02279-s001.zip › File S1.pdf]

Supplementary File S1

Curcumin in Atherogenic Dyslipidemia: Linking Preclinical Mechanistic Insights to Clinical Outcomes

Kamil Brodziński, Justyna Juszczyńska, Joanna Karbowska and Zdzislaw Kochan

Supplementary Table S1. Targeted search strategies for identification and selection of mechanistic and preclinical evidence.

Mechanistic Domain	Example Terms Combined with Curcumin Block
Absorption and bioavailability	absorption OR bioavailability OR intestinal uptake OR permeability OR transport
Metabolism	metabolism OR glucuronidation OR sulfation OR reduction
Cytochrome P450	"cytochrome P450"
Intestinal cholesterol uptake	NPC1L1 OR "cholesterol absorption" OR "intestinal cholesterol uptake"
Hepatic lipid metabolism	"hepatic lipid metabolism" OR lipogenesis OR "fatty acid oxidation"
MicroRNAs	microRNA OR miRNA
SIRT1 and PPARα	SIRT1 OR PPARA OR PPARα
ChREBP and SREBP-1	ChREBP OR SREBP-1 OR SREBF1
SREBP-2 and cholesterol synthesis	SREBP-2 OR SREBF2 OR HMGCR OR "cholesterol synthesis"
Bile acid metabolism	"bile acid" OR CYP7A1
