# Supplementary material for: Curcumin in Atherogenic Dyslipidemia: Linking Preclinical Mechanistic Insights to Clinical Outcomes
Source: Nutrients. 2026 Jul 11;18(14):2279. doi: 10.3390/nu18142279 (PMC13414635; doi:10.3390/nu18142279)
Supplement: Supplementary file 1 [file nutrients-18-02279-s001.zip › File S2.pdf]

Supplementary File S2

Curcumin in Atherogenic Dyslipidemia: Linking Preclinical Mechanistic Insights to Clinical Outcomes

Kamil Brodziński, Justyna Juszczyńska, Joanna Karbowska and Zdzislaw Kochan

Supplementary Table S2. Curcumin-induced modulation of cytochrome P450 isoforms and their functions.

CYP Isoform	Function	Modulation by Curcumin	References
CYP1A2	This enzyme is expressed predominantly in the liver and is involved in the metabolism of caffeine, theophylline, clozapine, olanzapine, and several other xenobiotics and endogenous compounds.	In a cell-based model conducted in a controlled laboratory setting, curcumin was found to impede the activity of CYP1A2. If such inhibition is found to be clinically relevant, it has the potential to reduce clearance and increase systemic exposure of CYP1A2 substrates. Nevertheless, the existence of direct evidence demonstrating clinically significant interactions in humans remains to be established.	[1,2]
CYP2B6	It has been demonstrated to contribute to the metabolism of several pharmaceutical agents, including bupropion, efavirenz, cyclophosphamide, ketamine, methadone, and propofol.	Curcuminoid extracts were found to inhibit CYP2B6-mediated metabolism in vitro. This could result in alterations to the exposure or activation of CYP2B6 substrates. The direction of the clinical effect is substrate dependent; inhibition may increase exposure to active drugs but reduce activation of prodrugs, such as cyclophosphamide.	[3,4]
CYP2C9	It has been demonstrated to metabolize several clinically significant drugs, including warfarin, phenytoin, certain non-steroidal anti-inflammatory drugs, and sulfonylureas. The activity of this enzyme is also influenced by genetic variation.	In vitro studies have demonstrated that curcumin and related curcuminoids exhibit inhibitory effects on CYP2C9 activity. In the instance of this occurrence in a living organism, a diminished clearance of CYP2C9 substrates has the potential to elevate drug exposure and the likelihood of concentration-dependent adverse effects, including bleeding with warfarin, hypoglycemia with sulfonylureas, or toxicity with phenytoin. However, the clinical evidence remains limited.	[3,5,6]
CYP2D6	Processing many commonly prescribed drugs. Including antidepressants, analgesics, opioids and beta-blockers.	This enzyme has been demonstrated to metabolize a wide range of pharmaceutical agents, including antidepressants, antipsychotics, beta-blockers, antiarrhythmics, and certain opioids. Furthermore, it has been demonstrated to facilitate the activation of specific prodrugs, including codeine, to their respective active metabolites.	[7,8]
CYP3A4	It is a significant intestinal and hepatic drug-metabolizing enzyme that plays a crucial role in the clearance of a substantial proportion of commonly used medications. These include certain statins, calcium-channel blockers, im-	Curcumin and curcuminoid extracts have been shown to inhibit CYP3A4-mediated metabolism in vitro. In instances where this is clinically relevant, a decrease in CYP3A4 activity could lead to a reduction in clearance	[1,3,9]

	munosuppressants, benzodiazepines, and other xenobiotics.	and an increase in systemic exposure of substrates that are susceptible to this effect. However, the magnitude and clinical significance of this interaction after oral curcumin supplementation remain uncertain.	
CYP17A1	This enzyme catalyzes the 17α-hydroxylase and 17,20-lyase reactions, which are essential for the biosynthesis of glucocorticoids and sex steroids, including androgen precursors such as dehydroepiandrosterone.	In experimental systems, curcumin was found to inhibit both 17α-hydroxylase and 17,20-lyase activities, suggesting its ability to reduce androgen synthesis. It is important to note that these findings are preclinical, and their relevance to curcumin supplementation in humans remains uncertain.	[10,11]
CYP19A1	This enzyme, known as aromatase, facilitates the conversion of C19 androgens, such as testosterone and androstenedione, into C18 estrogens.	In experimental models, curcumin demonstrated a concentration-dependent inhibition of aromatase activity, though its effectiveness was lower compared to established aromatase inhibitors. The clinical relevance of this effect remains to be elucidated.	[10,12,13]
CYP21A2	The enzyme is a steroid 21-hydroxylase required for adrenal corticosteroid synthesis. It converts progesterone and 17-hydroxyprogesterone into precursors of mineralocorticoids and glucocorticoids.	In experimental systems, curcumin was found to exhibit a modest inhibitory effect on CYP21A2 activity. In principle, substantial inhibition has the potential to interfere with corticosteroid biosynthesis. However, the available evidence is preclinical and does not establish a clinically significant effect in humans.	[10,14]

Table created by the authors (K.B., J.J. and Z.K.) based on data from the studies cited in each row (see "References" column). Note: Most reported effects of curcumin on CYP enzymes derive from in vitro, cell-based, or computational studies. They indicate the potential for interactions but do not establish clinically relevant inhibition at customary supplemental doses.

References

1. Sasaki, T.; Sato, Y.; Kumagai, T.; Yoshinari, K.; Nagata, K. Effect of Health Foods on Cytochrome P450-Mediated Drug Metabolism. *J Pharm Health Care Sci* **2017**, *3*, 14. https://doi.org/10.1186/s40780-017-0083-x.
2. UniProt CYP1A2 Cytochrome P450 1A2 Available online: https://www.uniprot.org/uniprotkb/P05177/entry (accessed on 24 February 2026).
3. Volak, L.P.; Ghirmai, S.; Cashman, J.R.; Court, M.H. Curcuminoids Inhibit Multiple Human Cytochromes P450, UDP-Glucuronosyltransferase, and Sulfotransferase Enzymes, Whereas Piperine Is a Relatively Selective CYP3A4 Inhibitor. *Drug Metab Dispos* **2008**, *36*, 1594–1605. https://doi.org/10.1124/dmd.108.020552.
4. UniProt CYP2B6 Cytochrome P450 2B6 Available online: https://www.uniprot.org/uniprotkb/P20813/entry#expression (accessed on 24 February 2026).
5. Daly, A.K.; Rettie, A.E.; Fowler, D.M.; Miners, J.O. Pharmacogenomics of CYP2C9: Functional and Clinical Considerations. *J Pers Med* **2017**, *8*, 1. https://doi.org/10.3390/jpm8010001.
6. UniProt CYP2C9 Cytochrome P450 2C9 Available online: https://www.uniprot.org/uniprotkb/P11712/entry (accessed on 24 February 2026).
7. Kane, M. CYP2D6 Overview: Allele and Phenotype Frequencies. In *Medical Genetics Summaries [Internet]*; National Center for Biotechnology Information (US), 2025.

8. UniProt CYP2D6 Cytochrome P450 2D6 Available online: https://www.uniprot.org/uniprotkb/P10635/entry (accessed on 24 February 2026).
9. UniProt CYP3A4 Cytochrome P450 3A4 Available online: https://www.uniprot.org/uniprotkb/P08684/entry (accessed on 24 February 2026).
10. Rodríguez Castaño, P.; Parween, S.; Pandey, A.V. Bioactivity of Curcumin on the Cytochrome P450 Enzymes of the Steroidogenic Pathway. *Int J Mol Sci* **2019**, *20*, 4606. https://doi.org/10.3390/ijms20184606.
11. UniProt CYP17A1 Steroid 17-Alpha-Hydroxylase/17,20 Lyase Available online: https://www.uniprot.org/uniprotkb/P05093/entry (accessed on 25 February 2026).
12. Lee, Y.-B.; Kim, C.; Hong, J.; Kim, D. Molecular Insights into CYP19A1 Mutations and Their Role in Estrogen Production. *Arch Biochem Biophys* **2025**, *772*, 110573. https://doi.org/10.1016/j.abb.2025.110573.
13. UniProt CYP19A1 Aromatase Available online: https://www.uniprot.org/uniprotkb/P11511/entry (accessed on 25 February 2026).
14. UniProt CYP21A2 Steroid 21-Hydroxylase Available online: https://www.uniprot.org/uniprotkb/P08686/entry (accessed on 25 February 2026).
